# Influence of Chronological Age, Anthropometric Characteristics and Biological Maturity on Eccentric Knee Flexion Strength During the Nordic Hamstring Exercise in Female International Youth Soccer Players

**DOI:** 10.1002/ejsc.70135

**Published:** 2026-02-20

**Authors:** Jack T. Hickey, Tommy R. Lundberg, Cian Sweeney, Áine MacNamara, Liam Sweeney

**Affiliations:** ^1^ Department of Sport Science and Nutrition Maynooth University Kildare Ireland; ^2^ Sports Performance, Recovery, Injury and New Technologies (SPRINT) Research Centre Australian Catholic University Melbourne Australia; ^3^ Division of Clinical Physiology Department of Laboratory Medicine ANA Futura Karolinska Insitutet Huddinge Sweden; ^4^ Unit of Clinical Physiology Karolinska University Hospital Stockholm Sweden; ^5^ School of Science and Technology Nottingham Trent University Nottingham UK; ^6^ Leicester Diabetes Centre Leicester General Hospital, University Hospitals of Leicester NHS Trust Leicester UK; ^7^ Diabetes Research Centre College of Life Sciences, University of Leicester Leicester UK; ^8^ School of Health and Human Performance Faculty of Science and Health Dublin City University Dublin Ireland

**Keywords:** athlete development, body mass, growth, height, performance test

## Abstract

This study aimed to investigate the influence of chronological age, anthropometric characteristics and biological maturity on eccentric knee flexion strength during the Nordic hamstring exercise in female international youth soccer players. We included 50 under‐15‐year‐old (chronological age = 14.1 ± 0.4 years) and 31 under‐16‐year‐old (chronological age = 15.0 ± 0.5 years) female international youth soccer players as participants in this study. We measured each participant's body mass, height and biological maturity expressed as the percentage of predicted adult height (PAH%). Each participant performed three maximal effort repetitions of the Nordic hamstring exercise on a field‐based testing device (NordBord, VALD Performance, Brisbane, Australia) to measure their eccentric knee flexion strength. We used linear regression to investigate individual associations between eccentric knee flexion strength and each predictor variable (body mass, height, chronological age and PAH%). In addition, a partial least squares (PLS) regression model was developed to predict the average eccentric knee flexion strength. Eccentric knee flexion strength had statistically significant associations with body mass (*p* < 0.01 and *R* ^2^ = 0.34), PAH% (*p* < 0.01 and *R* ^2^ = 0.18) and chronological age (*p* = 0.03 and *R* ^2^ = 0.06) but not with height (*p* = 0.11 and *R* ^2^ = 0.03). The 2‐component PLS model explained 36% of variance in eccentric knee flexion strength, with body mass and PAH% the most influential predictors. Body mass and biological maturation status should be considered when interpreting eccentric knee flexion strength testing results during the Nordic hamstring exercise in female youth soccer players as these factors appear more influential than chronological age or height in determining test performance.

## Highlights

- Body mass and biological maturity should be considered when interpreting Nordic hamstring exercise strength testing results in female youth soccer players
- Despite having a statistically significant association with eccentric knee flexion strength in this study, chronological age has less influence than body mass and biological maturity on Nordic hamstring exercise strength testing results
- Our findings highlight relevant participant characteristics to consider when interpreting Nordic hamstring exercise strength testing results in youth female soccer players

## 1 Introduction

The Nordic hamstring exercise (NHE) is widely used to test the eccentric knee flexion strength of athletes (Claudino et al. 2021). This test's popularity has grown following evidence that low levels of eccentric knee flexion strength during the NHE can increase hamstring injury risk (Opar et al. 2015; Timmins, Bourne, Shield, Williams, Lorenzen, and Opar 2016b) and persist following anterior cruciate ligament injury (Collings et al. 2021; Högberg et al. 2022, 2024; Timmins, Bourne, Shield, Williams, Lorenzen, and Opar 2016a). To appropriately interpret the results of this common test, practitioners must understand how measures of eccentric knee flexion strength during the NHE can be influenced by various participant characteristics.

In male athletes, body mass has consistently been shown to have a positive association with eccentric knee flexion strength during the NHE (Buchheit et al. 2016; Capaverde et al. 2024; Jeanguyot et al. 2023). Chronological age of youth athletes and measures of their biological maturity have also been associated with eccentric knee flexion strength during the NHE (Franchi et al. 2019; Jeanguyot et al. 2023; Kiers et al. 2021). Youth athletes of the same chronological age can vary substantially in both their body mass and biological maturity, with differences of up to 6 years in skeletal age and somatic growth observed between age‐matched peers (Borms 1986; Johnson 2015). Advanced biological maturation confers significant changes in the neuromuscular system, which can influence strength in youth athletes (Malina et al. 2004). However, apart from two studies in alpine skiing (Franchi et al. 2019; Kiers et al. 2021), the potential influence of these factors on eccentric knee flexion strength during the NHE in female youth athletes has not been investigated.

Female youth athlete participation in soccer is increasing around the world, with more opportunities for girls to progress through talent pathways and academies to professional teams (Emmonds et al. 2020; M Taylor et al. 2012). Practitioners working in soccer report that the NHE is more commonly used in female academies than male academies or senior professional teams from around the world (McQuilliam et al. 2022). Despite widespread implementation of the NHE in female youth soccer (Bandak et al. 2024), the potential influence of anthropometric characteristics, biological maturity and chronological age on eccentric knee flexion strength during the NHE remains unknown in this population. Addressing this evidence gap may help practitioners interpret results of eccentric knee flexion strength testing during the NHE in this context, while helping to address the broader underrepresentation of female participants in youth athlete research (Curran et al. 2019).

The aim of this study was to investigate the influence of body mass, height, chronological age and biological maturity on eccentric knee flexion strength measured during the NHE in female international youth soccer players.

## 2 Materials and Methods

### 2.1 Research Context

The Football Association of Ireland (FAI) is the National Governing Body for soccer in Ireland. As part of the FAI's national/international talent development programme for girls, at the under‐15‐year‐old (U15) age group, those players considered the highest performing nationally are selected into the FAI's U15 National Academy. Players selected into this U15 National Academy are exposed to increased developmental provision in preparation for international soccer. At the end of the year‐long U15 National Academy programme, the highest performing players, as perceived by the FAI, are eligible for selection into the Ireland under‐16‐year‐old (U16) international team.

### 2.2 Participants, Consent and Ethics

There were 84 female youth soccer players selected by the FAI in their U15 National Academy (*n* = 52) or U16 international team (*n* = 32) during the study period. Players had to be free from any current injury to participate in this study, which resulted in two players from the U15 squad and one player from the U16 squad being excluded from participation. Each of the remaining 81 players and one of their respective parents/guardians provided their informed assent and consent, respectively, to participate in this study. Ethical approval was granted by the (BSRESC‐2024‐37891) Research Ethics Committee (Maynooth University Research Ethics Committee).

### 2.3 Body Mass, Height and Chronological Age

The senior author measured each participant's body mass to the closest 0.1 kg using digital scales (SECA 807, Hamburg, Germany) and height to the closest 0.1 cm using a stadiometer (SECA 213, Hamburg, Germany), while participants wore socks, light training t‐shirts and shorts. Each participant's chronological age was expressed to the closest 0.1 years.

### 2.4 Biological Maturation

The biological maturation status of each participant was estimated using their body mass, chronological age and height, along with their parents' heights, to predict their adult height, as per the method described by Khamis and Roche (Khamis and Roche 1994). An online form was distributed by the FAI to the parents of each participant to self‐report their height, which was subsequently adjusted for overestimation (Epstein et al. 1995). Each participant's current height was then expressed as a percentage of their predicted adult height (PAH%), which provides an indication of biological maturity status. This method is based upon the presumption that biological maturation is more advanced in those closer to their predicted adult height (Khamis and Roche 1994).

### 2.5 Eccentric Knee Flexion Strength

Eccentric knee flexion strength was measured during the NHE using a field‐based testing device (NordBord, VALD Performance, Brisbane, Australia), which provides reliable measurement of peak force in youth female athletes (Franklin et al. 2024). Participants performed three submaximal warm‐up repetitions of the NHE at 50%, 70% and 90% of their perceived maximal effort. Following a 30‐s rest period, participants then performed three maximal effort repetitions of the NHE. Participants were instructed to control their fall by pulling up into the hooks of the testing device as hard as they could with strong verbal encouragement provided by the lead author. Left and right peak eccentric knee flexion force was measured in Newtons (N) during each maximal effort repetition, with the average peak force recorded across these three repetitions recorded for each limb. The between‐limb average of these values was subsequently calculated to generate a single measure of each participant's eccentric knee flexion strength for further analysis.

### 2.6 Statistical Analysis

Data were analysed using custom‐written code in the R programing language. Descriptive statistics (mean ± standard deviation) were reported for all participant characteristics within the sample. Mean ± standard deviation eccentric knee flexion strength measured during the NHE for the U15 and U16 squads were reported and compared using an un‐paired *t*‐test. We used linear regression to investigate individual associations between the outcome variable of eccentric knee flexion strength and each predictor variable (body mass, height, chronological age and PAH%). Statistical significance was set to *p* < 0.05 and *R* ^2^ values were reported.

In a further exploratory analysis of predictor‐outcome associations, partial least squares (PLS) regression was then used to model associations between eccentric knee flexion strength and four predictor variables (body mass, chronological age, height and PAH%). We used PLS regression to handle correlated predictor variables and moderate sample sizes. The predictor matrix (X) and outcome vector (Y) were standardised (mean = 0 and variance = 1) to ensure comparability between predictor variables. Predictor variances were checked to confirm nonzero variability. The optimal number of PLS components was determined using 10‐fold cross‐validation, selecting the number of components that minimised the root mean squared error of prediction (RMSEP). The final model was refitted with the selected components, and cross‐validated *R* ^2^ (Q^2^) was computed to assess predictive performance, alongside explained variance (*R* ^2^).

To explore the relative importance of each predictor variable on the outcome of eccentric knee flexion strength, variable importance in projection (VIP) scores were calculated to rank predictor importance, with VIP > 1 indicating high importance and VIP < 0.8 indicating low importance (Chong and Jun 2005). Given the exploratory nature of this analysis, we prioritised this interpretation of variable importance over predictive accuracy. The VIP formula used normalised loading weights, score variances and squared correlations between the outcome and PLS scores, ensuring robust differentiation across predictors. In addition, residual analysis was performed to identify under and overperformers in terms of eccentric knee flexion strength. This approach illustrates interpretation and practical use of the test results in combination with maturity‐related predictors to help facilitate performance profiling of youth female soccer players. Residuals were calculated as the difference between observed and predicted eccentric knee flexion strength during the NHE. Standardised residuals (z‐scores) were computed to classify players as overperformers (*z* > 1) or underperformers (*z* < −1). Results were visualised using score plots, VIP bar plots and predicted versus observed scatter plots with performance labels, generated in R studio.

## 3 Results

Participant characteristics within the sample are shown in Table 1.

**TABLE 1:** Mean ± standard deviation participant characteristics within the sample.

Variable	Participants (*n* = 81)
Body mass	57.3 ± 6.7 kg
Height	164.8 ± 5.6 cm
Chronological age	14.5 ± 0.6 years
Percentage of predicted adult height	98.5 ± 1.0%

**FIGURE 1:**
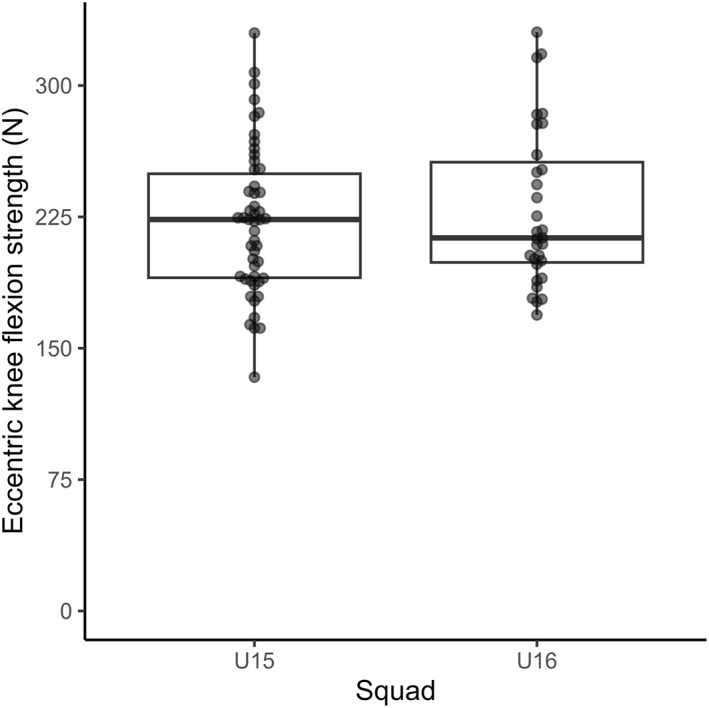
Eccentric knee flexion strength (N) measured during the Nordic hamstring exercise (y‐axis) within the under‐15‐year‐old (U15) and under‐16‐year‐old (U16) squads (x‐axis).

Mean ± standard deviation eccentric knee flexion strength was 223 ± 42 N in the U15 squad and 229 ± 45 N in the U16 squad, which was not a statistically significant difference (*p* = 0.52, Figure 1).

**FIGURE 2:**
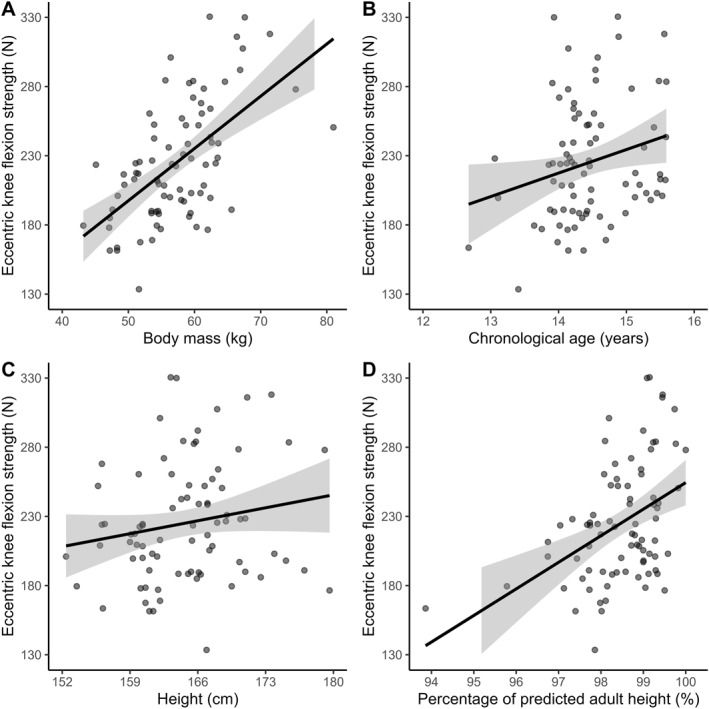
Eccentric knee flexion strength (N) measured during the Nordic hamstring exercise (y‐axis) and associations with each predictor variable (x‐axis) including body mass (A), chronological age (B), height (C) and percentage of predicted adult height (D).

Body mass had a statistically significant (*p* < 0.01) and positive association (*R* ^2^ = 0.34) with eccentric knee flexion strength (Figure 2A). Chronological age had a statistically significant (*p* = 0.03) and positive association (*R* ^2^ = 0.06) with eccentric knee flexion strength (Figure 2B). Height was not significantly (*p* = 0.11) associated (*R* ^2^ = 0.03) with eccentric knee flexion strength (Figure 2C). PAH% had a statistically significant (*p* < 0.01) and positive association (*R* ^2^ = 0.18) with eccentric knee flexion strength (Figure 2D).

**FIGURE 3:**
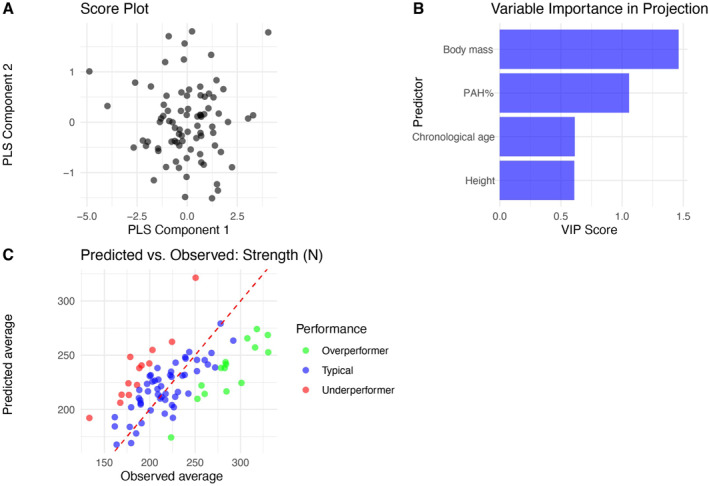
Score plot for partial least squares (PLS) model components 1 and 2 (A), variable of importance in projection (VIP) score for each predictor variable (B) and predicted versus observed eccentric knee flexion strength measured during the Nordic hamstring exercise (C).

The exploratory PLS model for eccentric knee flexion strength utilised two components, which were selected based on minimizing RMSEP (RMSEP values: intercept = 1.006, 1 component = 0.867, 2 components = 0.821, 3 components = 0.823 and 4 components = 0.827). The model explained 36.1% of the variance in eccentric knee flexion strength (*R* ^2^ = 0.361 and averaged across components) but exhibited low predictive power (*Q* ^2^ = 0.156), which indicates that the model is better suited to exploring predictor‐outcome associations than for out‐of‐sample prediction. The score plot (Figure 3A) displayed sample distribution in the first two PLS components, revealing no extreme outliers. The VIP scores identified body mass (VIP = 1.462) and PAH% (VIP = 1.057) as the most influential predictors of eccentric knee flexion strength from our exploratory model, supporting their strong association with eccentric knee flexion strength performance noted in the linear regression analysis. Chronological age (VIP = 0.613) and height (VIP = 0.609) were less influential on eccentric knee flexion strength, with VIP scores below 0.8 suggesting minimal contribution to the exploratory model (Figure 3B). The predicted versus observed plot (Figure 3C) visually highlighted the classifications of under and overperformers based on the predictor variables.

## 4 Discussion

This study investigated the influence of body mass, height, chronological age and biological maturation on eccentric knee flexion strength during the NHE in female youth international soccer players. Body mass had the strongest influence on eccentric knee flexion strength during the NHE, followed by PAH%, as an estimation of biological maturation status. Although we found a statistically significant association between chronological age and eccentric knee flexion strength, this individual association was relatively weak and was not shown to be an important predictor variable in our PLS model. Height was not associated with eccentric knee flexion strength measured during the NHE. The PLS model largely confirmed the individual associations between eccentric knee flexion strength and each predictor variable, with body mass and PAH% being the most significant predictors, highlighting their influence on NHE performance in female youth international soccer players.

The consensus across the literature in the male context has been that body mass is the most significant determinant of absolute eccentric knee flexion strength during the NHE (Buchheit et al. 2016; Jeanguyot et al. 2023; Markovic et al. 2020). Our study provides confirmatory evidence that body mass is also the most significant predictor of eccentric knee flexion strength measured during the NHE in female youth soccer players. We suggest that greater skeletal muscle mass is primarily responsible for the higher levels of eccentric knee flexion strength observed in those with greater body mass rather than overall body mass per se. Our findings support this suggestion as the absolute eccentric knee flexion strength in female youth international soccer players reported in our study are ∼40 N lower than those reported in previous studies with chronologically age‐matched males (Jeanguyot et al. 2023; Markovic et al. 2020).

Biological maturation drives phenotypic divergence between the sexes during puberty, with increasing oestrogen levels primarily responsible for the increase in fat mass in females, whereas increased testosterone concentrations promote the increase in height and muscle mass in males (Lloyd et al. 2014; Malina et al. 2004). Considered together, these physiological sex differences can largely explain why the eccentric knee flexion strength values reported in our study are lower than those reported in male populations during the NHE (Jeanguyot et al. 2023). Thus, practitioners should be aware that although advanced biological maturation is generally advantageous for absolute lower body muscular strength in youth athletes (Lloyd et al. 2014), even after peak height velocity, this is to a much lesser extent the case in females.

More biologically mature females generally have greater lean muscle mass and total body mass compared to their less mature peers (Lloyd and Faigenbaum 2016). Of note, some females have been shown to experience the onset of the adolescent growth spurt as young as 9.5 years (Engebretson et al. 2010), and it is possible that female youth soccer players' eccentric knee flexion strength during the NHE may be influenced by biological maturation status at earlier stages of development, before the ages where fat mass increases substantially. Given that our sample were predominantly 2–3 years post peak height velocity, this is a limitation of our sample and an area that warrants further exploration.

Our findings compliment previously published research that found more biologically mature female youth soccer players typically produce greater force in isometric mid‐thigh pull tests (IMTP) relative to their aged‐matched peers (Emmonds et al. 2017, 2020). However, it should be noted that the NHE test for eccentric knee flexion strength used in this study differs from the IMTP in both contraction mode and joint movement involved. Our findings confirm what has previously been found in female youth alpine skiers, where eccentric knee flexor strength measured using the NHE was also significantly correlated with chronological age and biological maturation (Kiers et al. 2021). Despite a generally limited evidence‐base, our study provides confirmatory evidence to suggest that more biologically mature female youth athletes are typically stronger in tests of absolute lower body strength, albeit to a lesser extent than males.

When measures of absolute lower body strength are assessed in female youth athletes, practitioners should be aware of the differences in biological maturation status (e.g., PAH%) between players before comparing individuals. With a residual analysis, such as the one used in our study, underperforming players can be identified, which may help coaches design training measures for improved strength development. Overall, our findings support the broader scientific literature in boys and girls showing that lower body strength increases with chronological age, biological maturation and body mass (Emmonds et al. 2017; Emmonds et al. 2020; Jeanguyot et al. 2023). Practitioners working within female youth soccer should regularly monitor the anthropometric characteristics, biological maturation and physical performance of players, with an understanding that these variables are interrelated. It is important to be aware that absolute lower body strength is associated with biological maturation status, and advanced biological maturation is also associated with increased body mass, which has the strongest associations with lower body strength. These factors should be considered when evaluating youth soccer players' performance in tests for muscular strength. Further, it is acknowledged that muscular strength is advantageous for athletic performance, and thus, practitioners should seek to develop muscular strength throughout childhood and adolescence (Emmonds et al. 2020; Lloyd and Faigenbaum 2016).

The PLS model should be interpreted as exploratory rather than predictive. Although the model explained 36% of variance in eccentric knee flexion strength, the low cross‐validated predictive power (*Q* ^2^ = 0.156) indicates limited generalisability to new samples. Therefore, the VIP scores and associations should be viewed as hypothesis‐generating insights into maturity‐related determinants of strength rather than as a validated predictive tool for individual athletes. Future studies with larger independent samples are needed to validate these associations and develop robust prediction models.

We acknowledge that our data are specific to U15 and U16 female international youth soccer players and applying these findings to broader age groups where variations in maturation may be larger is limited. Noninvasive methods to estimate biological maturity status were used based upon American youth of European ancestry and not those of Irish nationality (Khamis and Roche 1994). Normative growth reference standards may differ between different nationalities and ethnicities (Sweeney et al. 2024). In addition, parental heights were self‐reported, which introduces considerable uncertainty to the estimation of biological maturity. Although parental self‐reported heights were adjusted for overestimation, this adjustment equation is based upon participant data from the United States of America (Epstein et al. 1995), which may be less valid when applied to parents of participants in our study. Although these limitations must be considered when interpreting the findings of our study, limited access to resources often necessitates this pragmatic approach to estimating biological maturity in the context of youth sport (Sweeney et al. 2024). In future research, if access to resources are not limited, skeletal x‐ray assessments are used as the gold‐standard assessment of biological maturity (Lloyd et al. 2014), which may address some of the limitations associated with this study.

We also acknowledge that the lever arm (i.e., distance from the knee joint to ankle fixation hook) was not measured during the NHE, which limits comparability of eccentric knee flexion strength between participants. Peak force measured at the ankle hooks of this field‐based testing device explains just over half of the variance in estimated internal hamstring muscle forces during the NHE (Ruan et al. 2021). Therefore, our measure of eccentric knee flexion strength should not be interpreted as an isolated muscular property but rather as a mechanical output influenced by a combination of internal muscle forces, anthropometry and mechanical leverage. For example, if two athletes have the same peak eccentric knee flexion force measured during the NHE, but different leg lengths, the athlete with shorter legs has to produce more internal hamstring muscle force to achieve this mechanical output. Finally, it is also important to appreciate that the NHE requires a level of technical competency and capacity to tolerate a high loading stimulus, which limits findings to athletes who have been given appropriate technical instruction (Jeanguyot et al. 2023).

## 5 Conclusion

This study has found that eccentric knee flexion strength measured during the NHE is associated with body mass, biological maturity and chronological age in female youth international soccer players. Body mass had the strongest association with eccentric knee flexion strength, followed by PAH%, as a measure of biological maturation status. We encourage further research of this nature to enhance our understanding of the influence of body mass and biological maturation on physical performance characteristics in younger female populations, where interindividual variation in growth and maturation is typically larger.

## Funding

This research was funded by the Sport Ireland Research Funding Scheme Allocation 2023.

## Ethics Statement

This study was approved by the Maynooth University Research Ethics Committee (BSRESC‐2024‐37891).

## Consent

All participants provided their informed written consent to participate.

## Conflicts of Interest

The authors declare no conflicts of interest.

## Data Availability

The data that support the findings of this study are available from the corresponding author upon reasonable request.
